# Vinblastine Resistance Is Associated with Nephronophthisis 3-Mediated Primary Cilia via Intraflagellar Transport Protein 88 and Apoptosis-Antagonizing Transcription Factor

**DOI:** 10.3390/ijms251910369

**Published:** 2024-09-26

**Authors:** Pham Xuan Thuy, Tae-Kyu Jang, Eun-Yi Moon

**Affiliations:** Department of Bioscience and Biotechnology, Sejong University, Seoul 05006, Republic of Korea; phamxuanthuy150894@gmail.com (P.X.T.); xorb9909@naver.com (T.-K.J.)

**Keywords:** vinblastine resistance, primary cilium, nephronophthisis 3, IFT88, AATF

## Abstract

Primary cilia (PC) are microtubule-based organelles that function as cellular antennae to sense and transduce extracellular signals. Nephronophthisis 3 (NPHP3) is localized in the inversin compartment of PC. Mutations in NPHP3 are associated with renal-hepatic-pancreatic dysplasia. In this study, we investigated whether vinblastine (VBL), a microtubule destabilizer, induces anticancer drug resistance through NPHP3-associated PC formation in HeLa human cervical cancer cells. A considerable increase in PC frequency was observed in HeLa cells under serum-deprived (SD) conditions, which led to the inhibition of VBL-induced cell death. VBL-resistant cells were established by repetitive treatments with VBL and showed an increase in PC frequency. NPHP3 expression was also increased by VBL treatment under serum starvation as well as in VBL-resistant cells. NPHP3 expression and PC-associated resistance were positively correlated with apoptosis-antagonizing transcription factor (AATF) and negatively correlated with inhibition of NPHP3. In addition, AATF-mediated NPHP3 expression is associated with PC formation via the regulation of intraflagellar transport protein 88 (IFT88). VBL resistance ability was reduced by treating with ciliobrevin A, a well-known ciliogenesis inhibitor. Collectively, cancer cell survival following VBL treatment is regulated by PC formation via AATF-mediated expression of IFT88 and NPHP3. Our data suggest that the activation of AATF and IFT88 could be a novel regulator to induce anticancer drug resistance through NPHP3-associated PC formation.

## 1. Introduction

Vinca alkaloids are natural products originally derived from the Madagascar periwinkle plant, Catharanthus roseus. The four major vinca alkaloids in clinical use are vinblastine (VBL), vincristine, vinorelbine, and vindesine [1]. Vinca alkaloids bound to microtubules form alternate spiral polymers and depolymerize the microtubules [2,3]. Cancer cell division is blocked by vinca alkaloids [4]. VBL is effective in numerous types of cancers, including melanoma, leukemia, Hodgkin’s lymphoma, non-small cell lung, bladder, brain, cervix, breast, and testicular cancers [5,6]. VBL shortens mitotic microtubules and suppresses cell growth [7]. However, following long-term use of vinca alkaloids, cancer cells may become resistant to their treatment [8].

Resistance to anticancer drugs is acquired through various means such as the alteration of drug targets or cell proliferation, the expression of drug pumps or detoxification mechanisms, a decrease in apoptosis susceptibility, or an increase in the ability to repair DNA damage [9]. Anticancer drug resistance is a major problem in the treatment of cancer patients [10]. Thus, novel targets or mechanisms are needed for successful approaches to cancer treatment.

Primary cilia (PC) are microtubule-based antenna-like organelles in most vertebrate cells. The intraflagellar transport protein (IFT) controls the bidirectional movement of particles along ciliary microtubules in cilium assembly and disassembly [11]. PC transduces various signals in embryonic development and tissue homeostasis. Abnormal PC causes development disorders and various human diseases, including a variety of cancer types [12,13,14]. PC influences cancer progression and development by sustaining tumor cell survival and growth in hypoxic microenvironments [15]. Many types of pre-malignant and invasive tumor cells show a defect in PC frequency or length [16,17,18]. The number and length of PC are increased in tumor cells resistant to kinase inhibitors [19]. Abnormal PC shapes are also observed in cancer cells with anticancer drug resistance [20]. Thus, PC formation could be an emerging mechanism of VBL-induced resistance.

Nephronophthisis 3 (NPHP3) is a ciliary protein localized in the basal body and centrioles of PC. NPHP3 is responsible for the ciliopathies caused by ciliary dysfunction such as autosomal recessive kidney disorder, the most frequent genetic disease of renal failure in children and young adults [21,22,23,24]. NPHP3 plays a role in primary cilium length control in mouse kidney epithelial cells [25] and the zebrafish Kupffer’s vesicle [26]. In addition, NPHP3 regulates PC formation through interaction with thymosin beta-4 (Tβ4), which may lead to tumorigenesis with inappropriate regulation of Tβ4 and/or NPHP3 expression [27]. NPHP3 also controls cancer cell viability via PC formation under serum-deprived (SD) conditions [28]. However, little information has been reported about the role of PC formation in VBL-induced resistance.

The aim of this study was to investigate the involvement of PC formation in VBL-induced resistance under SD conditions in the absence of fetal bovine serum (FBS) using HeLa cervical tumor cells. We examined whether induction of VBL resistance could result from NPHP3-mediated PC formation via apoptosis-antagonizing transcription factor (AATF) and intraflagellar transport protein 88 (IFT88). Data showed that cell death was attenuated by VBL treatment under SD conditions, which is similar to VBL resistance as compared to the condition in the presence of FBS. Data also showed that VBL treatment induced the expression of NPHP3 and IFT88, which was regulated by AATF, suggesting that PC formation by NPHP3 might regulate VBL resistance through AATF activation and IFT88 induction. These findings suggest that AATF and IFT88 could be novel parameters to control anticancer drug resistance through NPHP3-mediated PC formation.

## 2. Results

### 2.1. Vinblastine Resistance Is Associated with PC Formation under Serum Starvation

**Figure 1:**
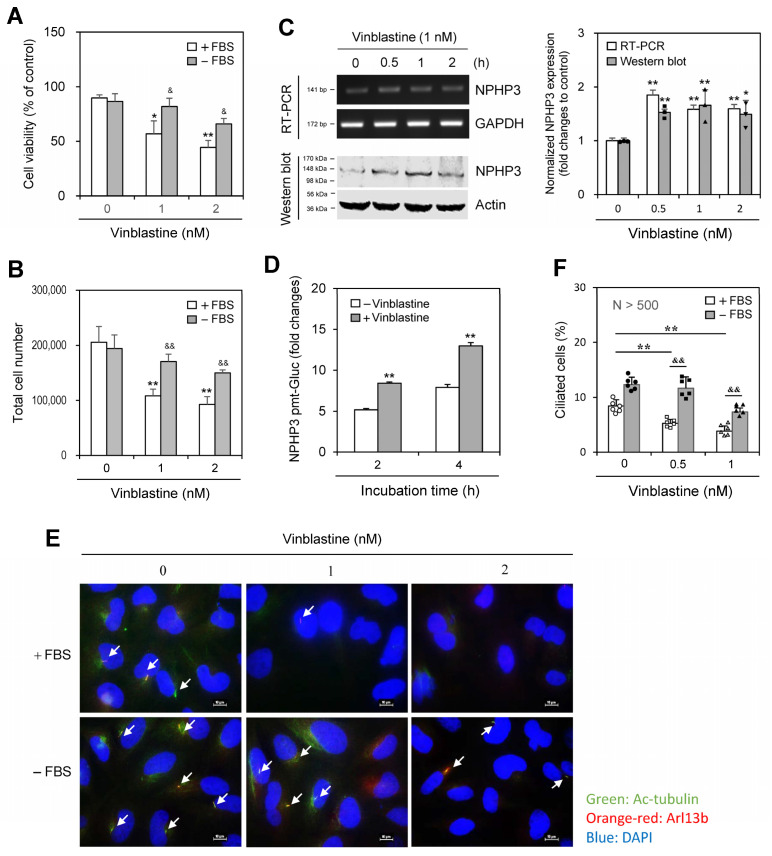
The anticancer effect of vinblastine (VBL) decreased under serum-deprived (SD) conditions and was associated with primary cilium formation and NPHP3 expression. (**A**–**C**) HeLa cells were incubated with various VBL concentrations in the presence or absence of fetal bovine serum (FBS) for 24 h. Cell viability was measured by trypan blue exclusion assay (**A**). Cell number was counted by using a hemocytometer (**B**). Total RNA in the cells incubated under SD condition was prepared by using NucleoZOL^®^ and the expression level of NPHP3 was measured by RT-PCR (**C** left top). Cell lysates in the cells incubated under SD conditions were prepared and each protein was detected by western blot analysis (**C** left bottom). The density of NPHP3 amount in the VBL-treated group was quantitated with NIH image analysis software (version 1.54h) and normalized to control. Fold changes in NPHP3 were represented with a bar graph (**C** right). (**D**) HeLa cells were transfected with pEZX-PG02-NPHP3-promoter (pmt) Gaussia luciferase (Gluc) plasmid and incubated in the presence or the absence of FBS for up to 24 h. The activity of Gluc in cultured media was measured with a luminometer using a Gluc substrate. (**E**,**F**) HeLa cells were incubated with VBL in the presence or absence of FBS for 24 h. The cells were fixed and stained with antibodies against Ac-tubulin (green) and Arl13b (orange-red). The nucleus was stained with DAPI (blue). The primary cilium was observed and photographed at 400× or 1000× magnification with 40× or 100× objectives, respectively under a fluorescence microscope. The image with primary cilia at 1000× magnification is representative of ≈30 pictures. White arrows indicated primary cilia (**E**). The ciliated HeLa cells (n > 500 cells) in the presence (white) or absence (grey) of FBS were counted (**F**). Data were representative of four experiments. Processing (such as changing brightness and contrast) is applied equally to control across the entire image (**C** left, **E**). Data in bar graphs represents the means ± SEM. * *p* < 0.05, ** *p* < 0.01; significantly different from VBL-untreated control group in the presence of FBS (**A**,**B**,**C** right, **D**,**F**). & *p* < 0.05, && *p* < 0.01; significantly different from the control group in the presence of FBS at each concentration (**A**,**B**,**F**).

As many studies have reported that PC formation is induced by incubation with a low percentage of serum [29,30,31,32] and that NPHP3 regulates PC formation in HeLa cells [27], we investigated whether anticancer drug resistance is associated with PC formation and NPHP3 expression using HeLa cells. When cells were treated with various categories of anticancer drugs, including tubulin-dependent curcumin (10 μM), VBL (1 nM) or nocodazole (3.32 μM), and tubulin-independent trichostatin (1 μM), in the presence or absence of FBS, anticancer drug resistance was induced by VBL or nocodazole treatment under SD conditions in the absence of FBS. Decreased cell viability in the absence of FBS was inhibited by ≈50% and ≈30% with VBL and nocodazole treatment, respectively compared to each control in the presence of FBS (Supplementary Figure S1). Subsequently, we used more effective VBL in the rest of our studies. Decreased cell viability and total cell number were inhibited by ≈50% with 1 or 2 nM VBL treatment (Figure 1A,B). VBL treatment under SD conditions increased NPHP3 expression, which was determined by reverse transcription polymerase chain reaction (RT-PCR) (Figure 1C left top and right), western blot analysis (Figure 1C left bottom and right), and NPHP3 transcriptional activity (Figure 1D). VBL treatment also increased PC formation, which was determined by immunostaining (Figure 1E) and counting (Figure 1F). These results suggest that VBL resistance induction could be associated with NPHP3-mediated PC formation.

### 2.2. Repetitive Treatment with VBL Increased Drug Resistance, PC Formation, and NPHP3 Expression

**Figure 2:**
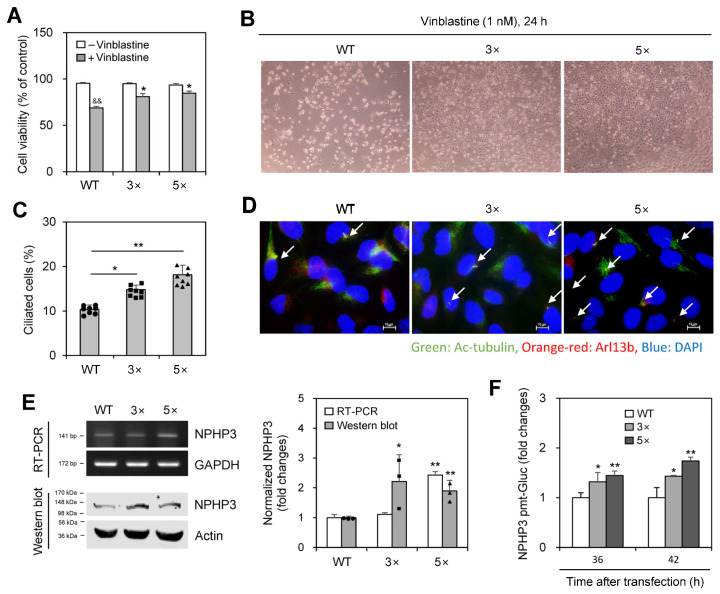
Decreased anticancer effect of vinblastine (VBL) on cells treated repeatedly with VBL was associated with primary cilium formation and NPHP3 expression. (**A**–**E**) Wildtype (WT) cells were treated with VBL once or repeatedly three (3×) to five (5×) times. Then each cell population was plated and treated with 1nM of VBL for 24 h. Cell viability was measured by the trypan blue exclusion assay (**A**). VBL-treated cells were observed under the bright-field microscope (**B**). The cells were fixed and stained with antibodies against Ac-tubulin (green) and Arl13b (orange-red). The nucleus was stained with DAPI (blue). The ciliated HeLa cells (n > 500 cells) were counted (**C**). The primary cilium was observed and photographed at 400× or 1000× magnification with 40× or 100× objectives, respectively under a fluorescence microscope. The image with primary cilia at 1000× magnification is representative of ≈30 pictures. White arrows indicated primary cilia (**D**). Total RNA was prepared by using NucleoZOL^®^ and the expression level of NPHP3 was measured by RT-PCR (**E** left top). Cell lysates were prepared and each protein was detected by western blot analysis (**E** left bottom). The density of NPHP3 amount in repeatedly (3× or 5×) VBL-treated group was quantitated with NIH image analysis software (version 1.54 h) and normalized to control. Fold changes in NPHP3 were represented with a bar graph (**E** right). (**F**) WT cells and VBL-treated cells repeatedly with VBL three (3×) or five (5×) times were transfected with pEZX-PG02-NPHP3-promoter (pmt) Gaussia luciferase (Gluc) plasmid and incubated in the presence or the absence of FBS for up to 24 h. The activity of Gluc in cultured media was measured with a luminometer using a Gluc substrate. Data were representative of four experiments. Processing (such as changing brightness and contrast) is applied equally to controls across the entire image (**C**,**E**). Data were representative of four experiments. Processing (such as changing brightness and contrast) is applied equally to control across the entire image (**B**,**D**). Data in bar graphs represents the means ± SEM (**A**,**C**,**E** right, **F**). ^&&^ *p* < 0.01; significantly different from the VBL-untreated control group (**A**). * *p* < 0.05, ** *p* < 0.01; significantly different from VBL-treated WT cells (**A**,**C**) or WT control cell (**E** right, **F**).

We examined whether PC formation is associated with the induction of VBL resistance. When wild-type (WT) HeLa cells were treated with VBL once or repeatedly three (3×) to five (5×) times, cell death was inhibited by repetitive treatments, with viability increasing to ≈80% and 85% in 3× and 5× VBL-treated cells, respectively, compared to ≈66% in single-treated WT control, which was determined by the trypan exclusion assay (Figure 2A,B). PC frequency, respectively, increased by repetitive treatments 1.4 and 1.8 times in 3× and 5× VBL-treated cells compared to WT control, which was determined by immunostaining (Figure 2C,D). Transcript and protein levels of NPHP3 also increased in 3× and 5× repeatedly VBL-treated cells compared to WT control, which was determined by RT-PCR (Figure 2E left top and right) and western blot analysis (Figure 2E left bottom and right), respectively, and confirmed by an increase in NPHP3-promoter activity (Figure 2F). These results suggest that PC formation might regulate VBL resistance induction.

### 2.3. Changes in NPHP3 Expression Modified Cell Death by Vinblastine

**Figure 3:**
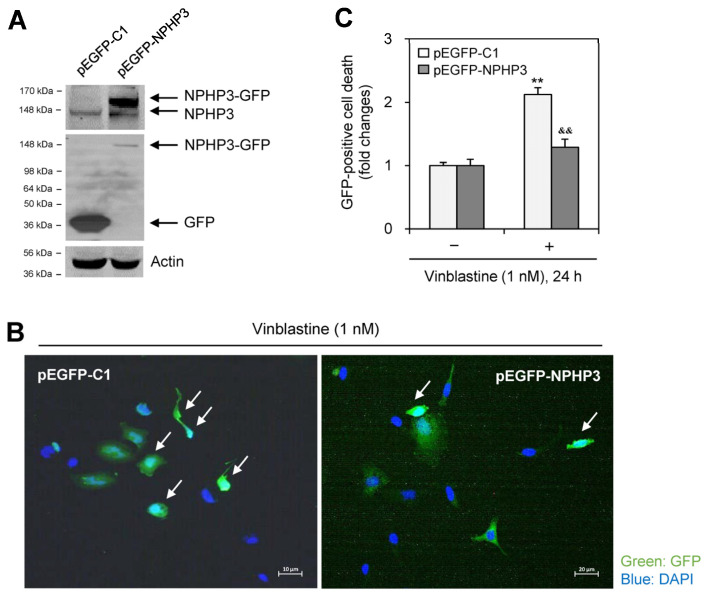
NPHP3 overexpression in HeLa cells reduced the death. (**A**–**C**) HeLa cells were transfected with pEGFP-C1 or pEGFP-HPHP3 for 36 h. Cell lysates were prepared and western blot analysis was performed to detect the expression of various proteins, NPHP3-GFP (**A** top), NPHP3 (**A** top and middle), GFP (**A** middle) and tubulin (**A** bottom). Cells in each group were treated with 1 nM VBL in the presence of FBS for 24 h. Then, cells were fixed and permeabilized and the nucleus was stained with DAPI (blue). Apoptotic cell death out of GFP-positive cells was observed at 400× magnification with 40× objective under the fluorescence microscope (**B**,**C**). The image with GFP fluorescence is representative of ≈30 pictures. White arrows indicated the dying cells with nuclear condensation (**B**). Bar graph represents the relative cell death (**C**). Processing (such as changing brightness and contrast) is applied equally to control across the entire image (**A**,**B**). Data in a bar graph represents the means ±SEM. ** *p* < 0.01; significantly different from the VBL-untreated control group. ^&&^ *p* < 0.01; significantly different from the VBL-treated group (**C**).

We tested whether NPHP3 expression is associated with the induction of VBL resistance. When NPHP3 was overexpressed by transfection with pEGFP-NPHP3 plasmid in the presence of FBS (Figure 3A), cell death as judged by nuclear condensation was reduced in the GFP-positive cell population (Figure 3B,C). These data indicate that NPHP3 increased drug resistance by decreasing cancer cell responsiveness to VBL.

**Figure 4:**
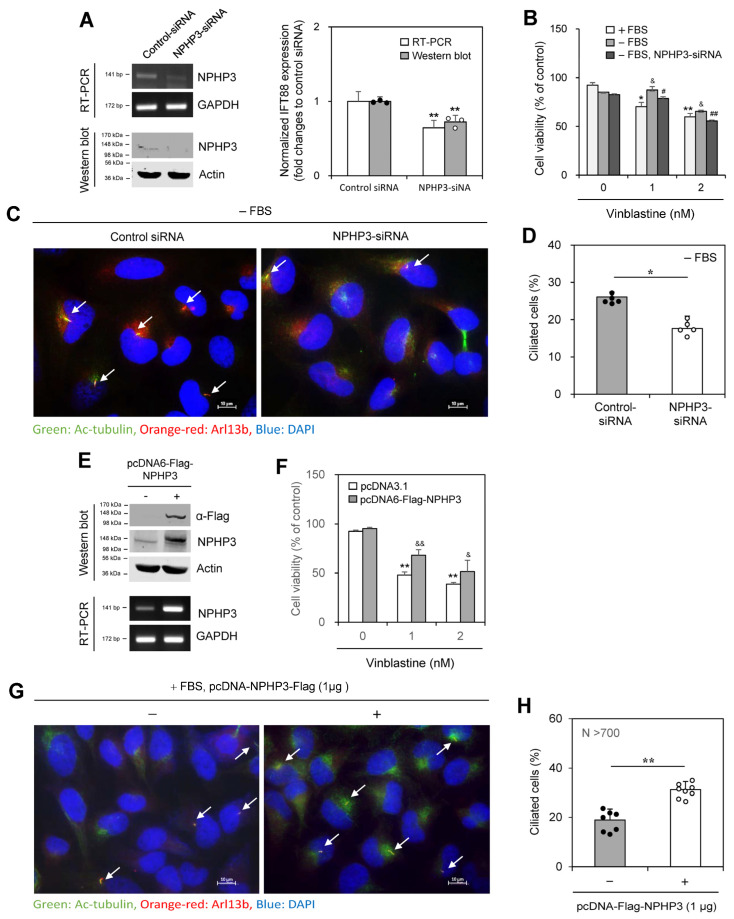
Cell death was decreased or increased by the transfection of HeLa cells with NPHP3-siRNA or pcDNA-Flag-NPHP3 plasmids, respectively. (**A**–**D**) HeLa cells were transfected with AccuTarget™ negative control siRNA (NC) or NPHP3-siRNA for 24 h. The expression level of NPHP3 transcripts was measured by RT-PCR (**A** left top). Cell lysates were prepared and NPHP3 protein was detected by western blot analysis (**A** left bottom). The density of NPHP3 amount in the NPHP3-siRNA-transfected group was quantitated with NIH image analysis software (version 1.54h) and normalized to control. Fold changes in NPHP3 were represented with a bar graph (**A** right). Cells were treated with 1 or 2 nM VBL for 24 h. Cell viability was measured by MTT assay (**B**). (**E**–**H**) HeLa cells were transfected with pcDNA3.1 or pcDNA6-Flag-NPHP3 for 24 h. Cell lysates were prepared and each protein was detected by western blot analysis (**E** top). The expression level of NPHP3 transcripts was measured by RT-PCR (**E** bottom). Cells were treated with 1 or 2 nM VBL for 24 h. Cell viability was measured by MTT assay (**F**). The cells were fixed and stained with antibodies against Ac-tubulin (green) and Arl13b (orange-red). The nucleus was stained with DAPI (blue). The primary cilium was observed and photographed at 400× or 1000× magnification with 40× or 100× objectives, respectively under a fluorescence microscope and indicated with white arrows. The image with primary cilia is the representative out of ≈30 pictures (**C**,**G**). The ciliated HeLa cells (n > 500 cells) in the presence (white) or absence (grey) of NPHP3-siRNA (**D**) or pcDNA6-Flag-NPHP3 (**H**) were counted. Data were representative of four experiments. Processing (such as changing brightness and contrast) is applied equally to control across the entire image (**A**,**C**,**E**,**G**). Data in bar graphs represents the means ± SEM (**A** right, **B**,**D**,**F**,**H**). * *p* < 0.05, ** *p* < 0.01; significantly different from control siRNA-treated group (**A** right, **D**) or VBL-untreated control group in the presence of FBS (**B**) or pcDNA3.1-transfected (**F**) or pcDNA6-Flag-NPHP3-untransfected (**H**) control group. & *p* < 0.05, && *p* < 0.01; significantly different from the control group in the presence of FBS (**B**) or pcDNA3.1-transfected (**F**) control group at each VBL concentration. # *p* < 0.05, ## *p* < 0.01; significantly different from NPHP3-siRNA-untreated group at each VBL concentration in the absence of FBS (**B**).

We next tested the effect of NPHP3 knockdown on PC formation. The inhibition of NPHP3 expression by NPHP3-siRNA was confirmed by RT-PCR (Figure 4A left top and right) and western blotting (Figure 4A left bottom and right). The cell viability of ≈70% by 1 nM VBL treatment in the presence of FBS was increased to ≈86% under SD conditions and then reduced to ≈77% by NPHP3-siRNA in the absence of FBS (Figure 4B). PC frequency was reduced by ≈33% by the knockdown of NPHP3 (Figure 4C,D). Overexpression of NPHP3 was confirmed by western blotting and RT-PCR (Figure 4E). The cell viability of ≈50% and ≈40% by 1 and 2 nM VBL treatment in the presence of FBS increased to ≈70% and ≈52% with NPHP3 overexpression (Figure 4F). NPHP3 overexpression approximately doubled the number of ciliated cells (Figure 4G,H). No change in primary cilium length was observed by NPHP3 overexpression (Supplementary Figure S2A). In addition, although SD conditions reduced cell proliferation compared to incubation in the presence of FBS, no change in cell proliferation was detected in the NPHP3-overexpressed group (Supplementary Figure S2B). Therefore, these data suggest that NPHP3 expression could modulate cell death by the anticancer agent, VBL, via PC formation in tumor cells.

### 2.4. Vinblastine Treatment Controls IFT88

**Figure 5:**
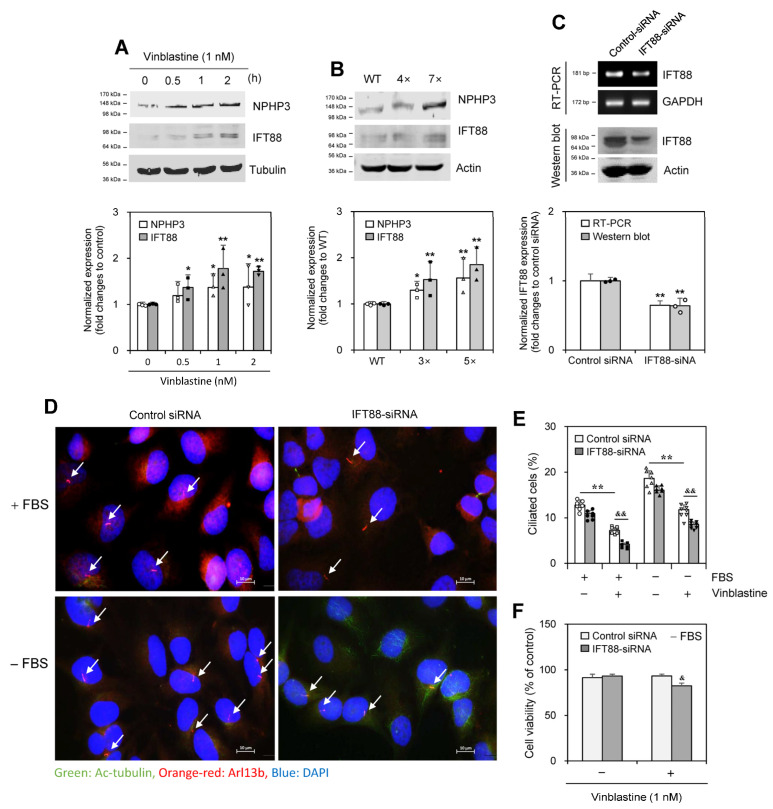
Intraflagellar transport protein 88 (IFT88) increased by the treatment with vinblastine (VBL) controlled primary cilium formation and cancer cell viability. (**A**,**B**) Wildtype (WT) HeLa cells were treated with 1 nM of VBL once (**A** top) or repeatedly four (4×) or seven (7×) times (**B** top). Cell lysates from each population were prepared and each protein was detected by western blot analysis (**A**,**B** top). The density of NPHP3 or IFT88 amount in each group was quantitated with NIH image analysis software (version 1.54h) and normalized to control. Fold changes in NPHP3 or IFT88 were represented with a bar graph (**A**–**B** bottom). (**C**–**F**) HeLa cells were transfected with AccuTarget™ negative control siRNA or IFT88-siRNA for 24 h. The expression level of IFT88 transcripts was measured by RT-PCR (**C** top upper). Cell lysates were prepared and IFT88 protein was detected by western blot analysis (**C** top lower). The density of IFT88 amount in each group was quantitated with NIH image analysis software (version 1.54h) and normalized to control. Fold changes in IFT88 were represented with a bar graph (**C** bottom). Cells were treated with 1 nM VBL for 24 h. The cells were fixed and stained with antibodies against Ac-tubulin (green) and Arl13b (orange-red). The nucleus was stained with DAPI (blue). The primary cilium was observed and photographed at 400× or 1000× magnification with 40× or 100× objectives, respectively under a fluorescence microscope. The image with primary cilia at 1000× magnification is representative of ≈30 pictures. White arrows indicated primary cilia (**D**). The ciliated HeLa cells (n > 500 cells) in the absence (white) or presence (grey) of IFT88-siRNA were counted (**E**). Cell viability incubated under serum deprivation was measured by MTT assay (**F**). Data were representative of four experiments. Processing (such as changing brightness and contrast) is applied equally to control across the entire image (**A**–**C** top, **D**). Data were representative of four experiments. Data in bar graphs represents the means ±SEM (**A**–**C** bottom, **E**, **F**). * *p* < 0.05, ** *p* < 0.01; significantly different from VBL-untreated control group in the presence of FBS (**A** bottom, **E**) or WT cells (**B** bottom) or control siRNA-treated group (**C** bottom) or VBL-untreated control group in the presence or absence of FBS (**E**). ^&^ *p* < 0.05, ^&&^ *p* < 0.01; significantly different from VBL-treated and control siRNA-treated control group in the presence or absence of FBS (**E**,**F**).

As IFT88, a ciliary maintenance protein, is one of the subunits of the IFT-B complex [33,34], we examined the ability of IFT88 to control PC formation in response to VBL treatment. IFT88 expression increased in response to VBL treatment for 0.5~2 h along with NPHP3 expression (Figure 5A). In addition, expression of IFT88 protein increased in HeLa cells treated with VBL once or repeatedly four (4×) to seven (7×) times (Figure 5B). IFT88 knockdown by IFT88-siRNA reduced IFT88 transcript (Figure 5C, upper top and lower) and protein (Figure 5C upper bottom and lower). IFT88 knockdown decreased PC frequency in VBL-untreated control cells (Figure 5D,E). IFT88 knockdown decreased PC frequency by ≈50% and 30% in the presence and absence of FBS, respectively, in VBL-treated cells (Figure 5E). Also, cell viability by 1 nM VBL treatment was reduced by ≈10% by the knockdown of IFT88 in the presence of FBS (Figure 5F). These results suggest that IFT88 could be associated with NPHP3 for PC formation in response to VBL treatment both in the presence of FBS and in SD conditions in the absence of FBS.

### 2.5. NPHP3 Controls IFT88 Expression

**Figure 6:**
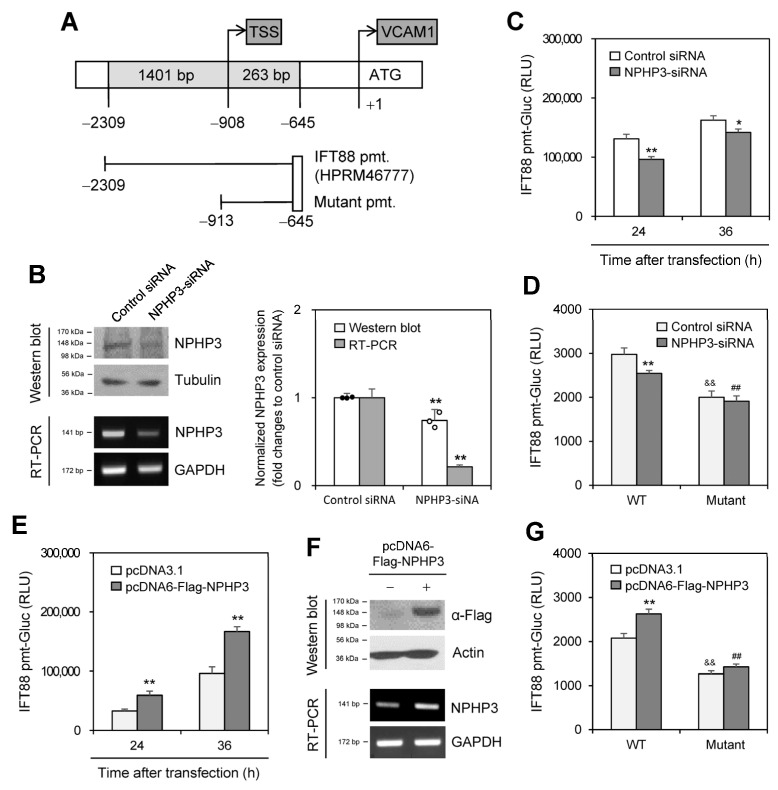
NPHP3 regulated intraflagellar transport protein 88 (IFT88) expression. (**A**) Pre-designed IFT88 promoter (HPRM46777) was obtained from GeneCopoeia Inc. The mutant IFT88 promoter (pmt) was prepared by the deletion of 1396 bp (−2308~−912). (**B**–**D**) AccuTarget™ negative control siRNA or IFT88-siRNA were co-transfected with pEZX-PG02-IFT88-wildtype (WT)-pmt-Gaussia luciferase (Gluc) plasmids (**C**,**D**) or pEZX-PG02-IFT88 mutant-pmt-Gluc plasmids (**D**) into HeLa cells. Cells were incubated for 24 h (**B**–**D**) or 36 h (**C**). Cell lysates were prepared and IFT88 protein was detected by western blot analysis (**B** left top). Expression level of NPHP3 transcripts was measured by RT-PCR (**B** left bottom). The density of NPHP3 amount in the NPHP3-siRNA-transfected group was quantitated with NIH image analysis software (version 1.54h) and normalized to control. Fold changes in NPHP3 were represented with a bar graph (**B** right). The activity of Gluc in cultured media was measured with a luminometer using a Gluc substrate. (**C**,**D**). (**E**–**G**) pcDNA3.1 or pcDNA6-Flag-NPHP3 plasmids were co-transfected into HeLa cells with pEZX-PG02-IFT88-WT-pmt-Gluc plasmids (**E**,**G**) or pEZX-PG02-IFT88 mutant pmt-Gluc plasmids (**G**). Cells were incubated for 24 h (**E**–**G**) or 36 h (**E**). Cell lysates were prepared and flag protein was detected by western blot analysis (**F**, top). The expression level of NPHP3 transcripts was measured by RT-PCR (**F** bottom). The activity of Gluc in cultured media was measured with a luminometer using a Gluc substrate. (**E**,**G**). Data were representative of four experiments. Processing (such as changing brightness and contrast) is applied equally to control across the entire image (**B** left, **F**). Data were representative of four experiments. Data in bar graphs represents the means ± SEM (**B** right, **C**,**D**,**E**,**G**). * *p* < 0.05, ** *p* < 0.01; significantly different from control siRNA-transfected group (**B** right, **C**,**D**) or pcDNA3.1-transfected group at each time point (**E**,**G**). ^&&^ *p* < 0.01; significantly different from WT IFT88-pmt plasmid-transfected and control siRNA-transfected (**D**) or pcDNA3.1 plasmid-transfected group (**G**). ^##^ *p* < 0.01; significantly different from WT IFT88-pmt plasmid-transfected and NPHP3-siRNA-transfected (**D**) or pcDNA6-Flag-NPHP3 plasmid-transfected group (**G**).

To confirm the effect of NPHP3 on IFT88 expression, we prepared an upstream-deleted mutant of the IFT88-promoter (Figure 6A). Then, we examined the effect of NPHP3 knockdown on IFT88 expression. The inhibition of NPHP3 expression by NPHP3-siRNA was confirmed by western blotting (Figure 6B left top and right) and RT-PCR (Figure 6B, left bottom and right). IFT88-promoter activity at 24 or 36 h was inhibited by ≈25% and ≈15%, respectively, by NPHP3 knockdown (Figure 6C). As shown in Figure 6D, IFT88-promoter activity decreased by ≈14% by NPHP3 knockdown and was further inhibited by ≈35% by the upstream deletion (−2309~−912) in the IFT88-promoter. When cells were co-transfected with IFT88-promoter and pcDNA6-Flag-NPHP3 plasmid, IFT88-promoter activity at 24 or 36 h was approximately doubled (Figure 6E) or increased by ≈15% (Figure 6G). NPHP3 expression was detected by western blotting and RT-PCR (Figure 6F). No increase in IFT88-promoter activity was observed in groups that were co-transfected with mutant IFT88-promoter and pcDNA6-Flag-NPHP3 plasmid (Figure 6G). These data suggest that NPHP3 upregulates IFT88 expression.

### 2.6. Apoptosis Antagonizing Transcription Factor Controls the Expression of NPHP3 and IFT88

**Figure 7:**
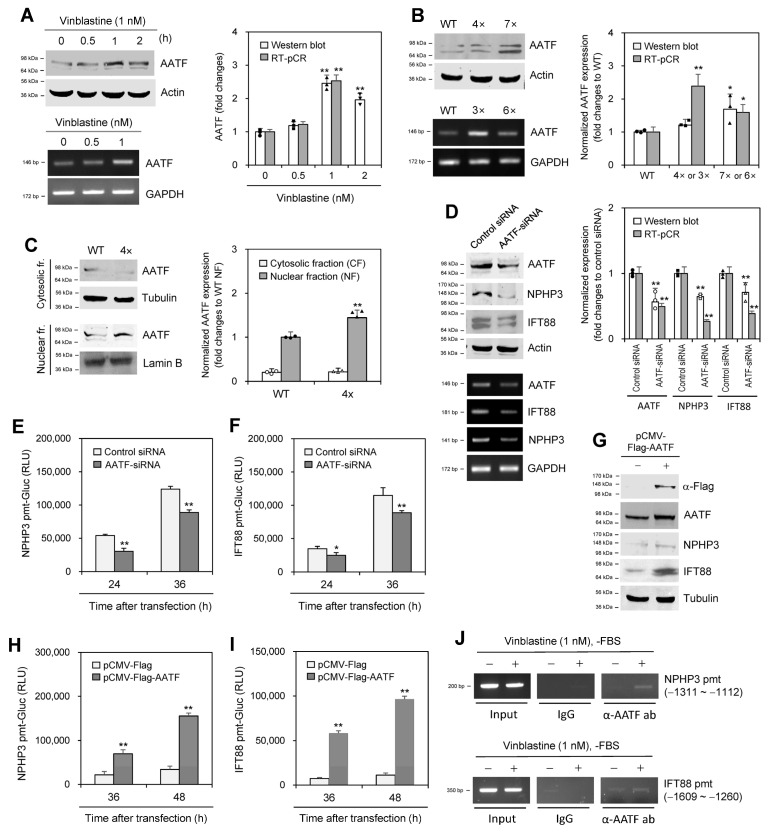
Apoptosis-antagonizing transcription factor (AATF) enhanced the expression of NPHP3 and IFT88. (**A**,**B**) Wildtype (WT) HeLa cells were treated with various concentrations of VBL once (**A**) or repeatedly with VBL three (3×) to seven (7×) times (**B**). Cell lysates from WT, 4× or 7× cells were prepared and each protein was detected by western blot analysis (**A**,**B** top). Expression level of AATF transcripts in WT, 3× or 6× cells was measured by RT-PCR (**A**,**B** bottom). (**C**) Cytosolic or nuclear fractions were separated from WT or 4× cells treated with VBL repeatedly four times. AATF protein was detected by western blot analysis. (**D**–**F**) AccuTarget™ negative control siRNA or AATF-siRNA were co-transfected into HeLa cells (**D**) with pEZX-PG02-NPHP3-pmt Gaussia luciferase (Gluc) plasmids (**E**) or pEZX-PG02-IFT88-pmt-Gluc plasmids (**F**). Cell lysates were prepared and each protein was detected by western blot analysis (**D** left top). The expression level of each transcript was measured by RT-PCR (**D** left bottom). The density of each protein and transcript was quantitated with NIH image analysis software (version 1.54h) and normalized to control. Fold changes in each protein were represented with a bar graph (**A**–**D** right). Cells were incubated for 24 or 36 h. The activity of Gluc in cultured media was measured with a luminometer using Gluc substrate (**E**,**F**). (**G**–**I**) pCMV-Flag or pCMV-Flag-AATF plasmids were co-transfected into HeLa cells with pEZX-PG02-NPHP3-pmt-Gluc plasmids (**H**) or pEZX-PG02-IFT88-pmt-Gluc plasmids (**I**). Cells were incubated for 24 or 36 h. Cell lysates were prepared and flag protein was detected by western blot analysis (**G**). The activity of Gluc in cultured media was measured with a luminometer using a Gluc substrate (**H**,**I**). (**J**) HeLa cells were incubated in the presence or the absence of 1 nM VBL for 1 h under serum-starved conditions. Cells were fixed with 10% formaldehyde. Their chromatin extracts were immunoprecipitated with anti-AATF antibodies (α-AATF ab). DNA fragments were subjected to PCR analysis using primer sets spanning each promoter (pmt) region. Data were representative of four experiments. Processing (such as changing brightness and contrast) is applied equally to control across the entire image (**A**–**E** left, **G**, **J**). Data in bar graphs represents the means ± SEM (**A**–**D** right, **E**,**F**,**G**,**I**). * *p* < 0.05, ** *p* < 0.01; significantly different from VBL-untreated control (**A**, right) or WT cells (**B** right) or nuclear fraction (**C** right) or control siRNA-transfected (**D** right, **E**,**F**) or pCMV-Flag plasmid-transfected (**H**,**I**) group.

We next investigated the molecular relevance controlling the expression of NPHP3 and IFT88. To find molecules that interacted with NPHP3, we performed a yeast two-hybrid (Y2H) screening assay using NPHP3 (691~1330) bait and a hybrid library of the human HeLa cell cDNA activation domain (AD) as prey. Candidate preys were initially selected, which satisfied the expression of three reporter genes. To reduce the possibility of picking up false positive candidates through the activation of reporter gene expression by AD fusion protein, we confirmed the interaction of each prey with NPHP3 (691~1330) bait (Supplementary Figure S3A,B). Then, 40 positive colonies were selected (Supplementary Figure S3C). Based on the findings in the yeast two-hybrid screening assay, we examined #34 colony apoptosis antagonizing transcription factor (AATF) in response to VBL treatment. AATF expression increased in response to VBL treatment for 0.5~2 h (Figure 7A). In addition, AATF expression increased in HeLa cells that were treated with VBL once or repeatedly three (3×) or four (4×) to six (6×) or seven (7×) times (Figure 7B). AATF protein was translocated into the nuclear fraction, which was higher in 4× cells compared to single-treated WT control cells (Figure 7C). This suggests that AATF expression might be transiently upregulated and downregulated in cancer cells treated repeatedly with VBL.

AATF knockdown by AATF-siRNA reduced AATF transcripts (Figure 7D left bottom and right) and protein (Figure 7D left bottom and right). AATF knockdown also decreased the expression of IFT88 and NPHP3 (Figure 7D). Additionally, AATF knockdown decreased the promoter activity of NPHP3 (Figure 7E) and IFT88 (Figure 7F). AATF overexpression by transfection with pCMV-Flag-AATF plasmids increased the expression of IFT88 and NPHP3 (Figure 7G). AATF overexpression also increased the promoter activity of NPHP3 (Figure 7H) and IFT88 (Figure 7I). Next, we examined AATF binding on each promoter for transcription of NPHP3 and IFT88 by chromatin immunoprecipitation (ChIP) assay. As shown in Figure 7J, the ChIP assay demonstrated increased AATF binding on the promoters of NPHP3 and IFT88 These results suggest that AATF could regulate NPHP3 and IFT88 expression for PC formation in response to VBL treatment.

### 2.7. Vinblastine-Mediated Cancer Cell Death Was Increased by the Inhibition of Ciliogenesis

**Figure 8:**
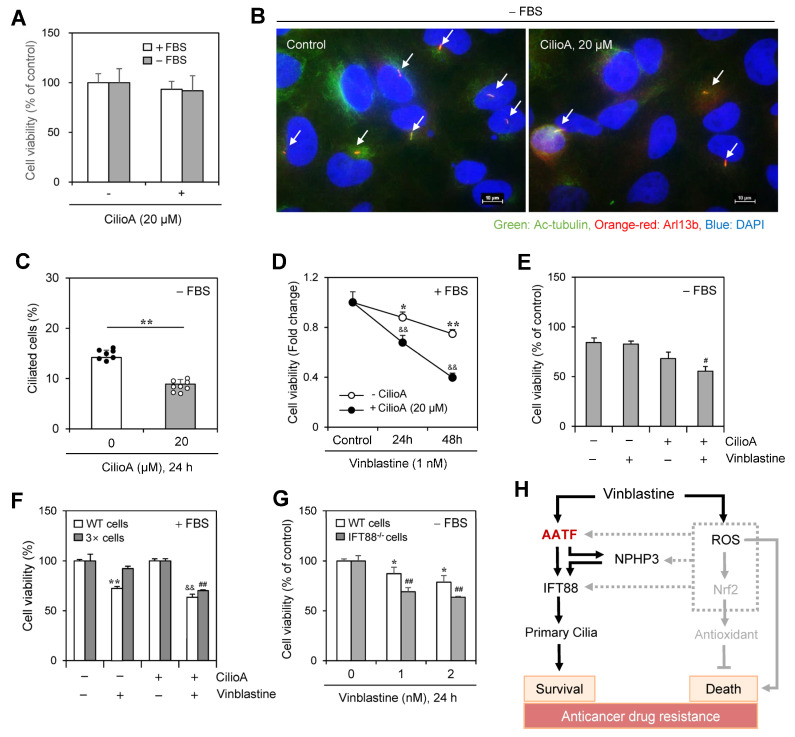
Inhibition of primary cilium formation by ciliobrevin A (CilioA) treatment enhanced vinblastine (VBL)-mediated cancer cell death. (**A**–**C**) HeLa cells were incubated in the absence or presence of CilioA. Cell viability was measured by CellTiter Glo luminescent assay (**A**). Cells incubated under serum-starved (SD) conditions were fixed and stained with antibodies against Ac-tubulin (green) and Arl13b (orange-red). The nucleus was stained with DAPI (blue). The primary cilium was observed and photographed at 400× or 1000× magnification with 40× or 100× objectives, respectively under a fluorescence microscope. The image with primary cilia at 1000× magnification is representative of ≈30 pictures. Processing (such as changing brightness and contrast) is applied equally to control across the entire image. White arrows indicated primary cilia (**B**). The ciliated HeLa cells (n > 500 cells) in the absence (white) or presence (grey) of CilioA were counted (**C**). (**D**–**G**) Wildtype (WT), 3× HeLa cells treated with VBL repeatedly three times or PC-deficient IFT88^−/−^ A375 cells were incubated with 1 nM VBL in the presence (**D**,**F**) or absence (**E**,**G**) of FBS for 24 h (**D**–**G**) or 48 h (**D**). Cell viability was measured by the trypan blue exclusion assay (**D**–**F**) or CellTiter Glo luminescent assay (**G**). Data were representative of four experiments. Data in the bar (**A**,**C**,**E**–**G**) or line (**D**) graphs represent the means ± SEM. * *p* < 0.05, ** *p* < 0.01; significantly different from the CilioA-untreated (**C**) or VBL-untreated (**D**,**F**,**G**) control group. ^&&^ *p* < 0.01; significantly different from CilioA-untreated and VBL-treated group in the presence of FBS (**D**,**F**). ^#^ *p* < 0.05, ^##^ *p* < 0.01; significantly different from CilioA-untreated and VBL-treated WT cell group in the absence of FBS (**E**) or 3× cell group in the presence of FBS (**F**) or VBL-treated WT cell group at each concentration (**G**). (**H**) Schematic mechanism for primary cilia formation by VBL-induced IFT88 through AATF and NPHP3 expression to regulate cancer cell survival and death. AATF regulated NPHP3 and IFT88 expression and primary cilium formation in HeLa human cervical cancer cells (solid line). ROS also upregulates NPHP3 expression through Nrf2 and unknown factors (grey dotted lines). Our findings were indicated by black solid lines. The pathway already known was indicated by grey solid lines.

We determined the number of cells with PC using ciliobrevin A (CilioA), a well-known ciliogenesis inhibitor. Little change in cell viability was observed by the treatment with CilioA (Figure 8A) and the frequency of PC in the absence of FBS was decreased ≈35% by CilioA treatment (Figure 8B,C). In addition, cell number was decreased ≈30~≈50% by co-treatment of VBL with CilioA in the presence of FBS (Figure 8D). Cancer cell viability was also decreased by ≈20% by co-treatment of VBL with CilioA in the absence of FBS (Figure 8E). Next, we examined the anticancer effect of VBL in cells treated with VBL repeatedly four times (4×). VBL-induced cell death increased by ≈25% in the incubation with CilioA (Figure 8F). In addition, since IFT88 is a critical ciliary maintenance protein in ciliogenesis [33,34], we examined the role of ciliogenesis on VBL-mediated cancer cell death using PC-negative IFT88^−/−^ cells. Then, we prepared PC-deficient IFT88^−/−^ A375 cells and examined VBL-mediated cancer cell death. A further ≈20% decrease in cancer cell viability was observed in PC-deficient IFT88^−/−^ A375 human melanoma cells compared to the WT control under SD conditions in the absence of FBS (Figure 8G). It explained that VBL-mediated cancer cell death was increased by the inhibition of ciliogenesis in another cell line except HeLa cells under SD conditions. These data confirmed that PC formation by VBL treatment regulates cancer cell survival and death, suggesting that cancer cell viability in response to VBL treatment could be regulated by PC formation through the AATF-NPHP3-IFT88 axis (Figure 8H).

## 3. Discussion

Anti-microtubule cancer drugs, including vinca alkaloids, are used in first- or second-line therapeutics alone or in combination [8]. Vinca alkaloids, including VBL, block the division of various types of cancers by binding to and shortening microtubules [2,3,4,5,6,7]. Following long-term use of vinca alkaloids, cancer cells become resistant to their treatment by the alteration of various cellular facets, including mutation or less expression of drug targets; overexpression of drug pumps; higher activity of drug detoxification mechanisms; low susceptibility to apoptosis; altered level of proliferation; and increased ability to repair DNA damage [8,9]. Anticancer drug resistance is a major unsolved therapeutic problem in treating cancer patients [10]. So, it is required to find novel targets or mechanisms for a successful approach to cancer treatment. PC in most vertebrate cells is microtubule-based antenna-like organelles and influences cancer progression and development [11,12,13,14,15]. Changes in the frequency, length, or shapes of PC are associated with anticancer drug resistance [16,17,18,19,20], indicating that PC formation could be an emerging mechanism of VBL-induced resistance. NPHP3, a ciliary protein, is responsible for various ciliopathies [21,22,23,24]. NPHP3 also controls cancer cell viability via PC formation under SD conditions [28]. However, little information has been reported on the role of PC formation on VBL-induced resistance. Here, we investigated whether NPHP3-mediated PC formation induces anticancer drug resistance against VBL under SD conditions in the absence of FBS. We found that PC formation by NPHP3 regulates VBL resistance through AATF activation and IFT88 induction. PC formation, cell viability, and NPHP expression were increased by VBL treatment under serum starvation (Figure 1) or by repetitive treatment with VBL (Figure 2). VBL-mediated cell death was controlled by the modification of NPHP3 expression (Figure 3 and Figure 4), demonstrating that NPHP3 expression could be increased by SD condition [28] as well as by VBL treatment (Figure 1, Figure 2, Figure 3 and Figure 4). VBL treatment controls IFT88 via NPHP3 expression (Figure 5 and Figure 6), which is associated with AATF binding to their promoters (Figure 7). VBL-mediated cancer cell death was increased by the inhibition of ciliogenesis (Figure 8). These data suggest that AATF and IFT88 could be novel parameters to control anticancer drug resistance through NPHP3-mediated PC formation.

The relationship between human cancer cell proliferation and PC formation remains contradictory and elusive. While some cancer cell division was inhibited by restoring PC formation [35,36], other cell proliferation was enhanced by a high frequency of PC [37], which is associated with the hedgehog signaling pathway [36,38,39]. Therefore, further studies are required to define the mechanism of action on ciliogenesis in tumor cells.

NPHP3 regulates PC formation via the interaction with Tβ4, an actin-sequestering protein, in HeLa cells [27]. PC is elongated by actin depolymerization and the enrichment of many actin-binding proteins inside the cilia [40]. Thus, there are several ways to interpret how NPHP3-mediated PC formation regulates VBL resistance under SD conditions (Figure 1, Figure 2, Figure 3 and Figure 4). The first possibility is that VBL resistance could be associated with the indirect effect of VBL on actin microfilaments as well as its direct effect on microtubules. The second possibility could be a direct effect of VBL regulating actin cytoskeletal structures in PC biogenesis. As the disruption of stress fibers increases cilia length and numbers [41,42,43], a third possibility is that VBL resistance could be established by the disruption or re-distribution of stress fibers [44]. Although further studies are required to investigate these possibilities, actin-associated changes could be a targetable mechanism in the induction of VBL resistance.

It is also important to determine whether NPHP3 localizes in the PC or elsewhere in the cell, as well as where NPHP3 is endogenously localized in resistant cells and how the localization differs between control and resistant cells. Previous reports showed that NPHP3 is a ciliary protein localized in the basal body and centrioles of PC [21,22,23,24]. In addition, NPHP3 was co-localized with Tβ4 at the cortical cell surface [27]. Thus, NPHP3 might be localized in the cortical cell surface of WT control cells and then transferred into the basal body and centrioles of PC in resistant cells. Further studies are required to clarify whether NPHP3 moves between the PC and the cortical cell surface when cancer cells become resistant.

We also investigated the mechanism of action in the interaction between NPHP3 and IFT88 expression. NPHP3 proteins are accumulated in the basal body, which is dependent on its coiled-coil domain. Then, the myristoylated NPHP3 complex enters the ciliary shaft [22,45] and localizes in the inversin compartment at the proximal PC segment [23]. PC formation is regulated by trafficking vesicular particles throughout the whole cell [46]. IFT controls the bidirectional movement of particles along ciliary microtubules in cilia assembly and disassembly [11]. IFT88 is a subunit of the IFT-B complex [33,34]. Our data showed that IFT88 expression is regulated by NPHP3 (Figure 5 and Figure 6), suggesting that NPHP3 could affect IFT88-mediated trafficking by the regulation of IFT88 expression.

To better understand the correlation between NPHP3, IFT88, and AATF proteins in PC formation, we examined the effect of AATF on the expression of NPHP3 and IFT88. High expression of AATF in high metastatic cell lines is cytoprotective against oxidative and apoptotic damage [47,48]. AATF regulates PC formation and modulates the DNA damage response [49]. NPHP3 expression and PC formation could be regulated by SD induction of hypoxia-inducible factor 1-alpha (HIF-1α) under normoxic conditions [28]. Our data indicate that the expression of AATF might be downregulated and its protein upregulation could be transient in ciliogenesis (Figure 7A,B) through NPHP3 and IFT88 expression (Figure 1, Figure 2, Figure 6 and Figure 7), allowing PC formation to return to basal levels. Our data also showed that the expression of NPHP3 and IFT88 was dependent on the binding of AATF to their promoters (Figure 7C). In addition, PC formation via AATF-mediated expression of NPHP3 and IFT88 could contribute to the induction of VBL resistance (Figure 8). These findings suggest that AATF might regulate PC formation and VBL resistance by controlling the expression of NPHP3 and IFT88 to appropriate levels.

In conclusion, while further studies are needed to define the mechanisms underlying AATF action, our results provide novel evidence of the effect of AATF on ciliogenesis and VBL resistance through the regulation of NPHP3 and IFT88 expression in HeLa cervical cancer cells. Our findings suggest that cooperation of NPHP3 and IFT88 may be required for PC formation via AATF binding to their promoters in tumor cells and underscore the need for further investigation of the involvement of AATF in the metastasis of ciliated tumor cells. Although we showed the results to reduce vinblastine resistance using siRNA for each protein, further studies are required to find possible inhibitors against those proteins that are overexpressed after treatment with vinblastine to reverse this drug resistance. Therefore, our results are biologically significant to provide some novel information about the physiological regulation of anticancer drug resistance in tumor cells and the pathological mechanism of PC-associated diseases.

## 4. Materials and Methods

### 4.1. Reagents, Plasmids, and siRNAs

4′,6-diamidno-2-phenylinole (DAPI, D9542) and 3-(4,5-Dimethyl-2-thiazolyl)-2,5-diphenyl-2H-tetrazolium bromide (MTT, M5655) were purchased from the Sigma Chemical Co. (St. Louis, MO, USA). Goat antibodies and rabbit antibodies, which are reactive with IFT88 (NBT100-2475) or AATF (ab39631), came from Novus Biologicals (Littleton, CO, USA) and Abcam (Cambridge, UK), respectively. Mouse antibodies, which are reactive with acetylated (Ac) tubulin (T7451) and β-tubulin (T4026), were from Sigma-Aldrich Co. (St. Louis, MO, USA). Mouse antibodies, which are reactive with actin (sc-376421), were from Santa Cruz Biotechnology, Inc. (Santa Cruz, CA, USA). Rabbit antibodies, which are reactive with NPHP3 (sc-134745) and GFP (sc-138), were from Santa Cruz Biotechnology, Inc. (Santa Cruz, CA, USA). Rabbit antibodies, which are reactive with Arl13b (17711-1-AP), were from Proteintech (Rosemont, IL, USA). Goat anti-rabbit IgG-Alexa 568 (A-11011), chicken anti-mouse IgG-Alexa 568 (A-21124), chicken anti-mouse IgG-Alexa 488 (A-21200), and lipofectamine^®^ 2000 transfection reagent were obtained from Invitrogen (Carlsbad, CA, USA). Polyethylenimine (PEI) was purchased from Polyscience (Warrington, PA, USA). IRDye 680RD Goat anti-Rabbit IgG (926-68071) and IRDye^®^ 680RD Donkey anti-Goat IgG (926-68074) were purchased from LI-COR Biotechnology (Lincoln, NE, USA). Except where indicated, all other materials are obtained from Sigma Chemical Co. (St. Louis, MO, USA).

pCDNA3.1 and pCMV-2B plasmids were kindly provided by Prof. Young-Joo Jang, College of Dentistry, Dankook University (Cheon-An, Republic of Korea). pEGFP-C2 plasmid was kindly provided by Prof. Mi-Ock Lee, College of Pharmacy, Seoul National University (Seoul, Republic of Korea). Flag-tagged pCDNA6-NPHP3 plasmid was kindly provided by Prof. Carsten Bergmann, Center for Human Genetics, Bioscientia (Ingelheim, Germany). pEGFP-C2-NPHP3 plasmids were generated by customer order for subcloning to Cosmo Genetech Co., Ltd. (Seoul, Republic of Korea).

Small interference (si) RNAs are customer-ordered by Bioneer (Daejeon, Republic of Korea). Sequences of siRNAs are as follows: NPHP3-siRNA with (sense: CUG UUG AAA UUC GAC AGA A (dTdT); anti-sense: UUC UGU CGA AUU UCA ACA G(dTdT)); IFT88-siRNA with (sense: CCG AAG CAC UUA ACA CUU A; anti-sense: UAA GUG UUA AGU GCU UCG G); AATF-siRNA with (sense: UAU GAG UUC UCG AAG GAG CUG; anti-sense: CAG CUC CUU CGA GAA CUC AUA). AccuTarget™ negative control siRNA (SN-1001) was also purchased from Bioneer (Daejeon, Republic of Korea).

### 4.2. Cell Culture

HeLa human cervical cancer cells (ATCC # CCL-2) and A375 human melanoma cells (ATCC # CRL-16192) were obtained from the Korea Research Institute of Bioscience and Biotechnology (KRIBB) cell bank (Daejeon, Republic of Korea). PC-deficient IFT88^−/−^ A375 human melanoma cells were kindly provided by Professor Ja-Hyun Koo, Seoul National University (Seoul, Republic of Korea). Cells were cultured in Dullecco’s modified Eagle’s medium (DMEM) supplemented with 10% FBS (GIBCO, Grand Island, NY, USA), 2 mM L-glutamine, 100 units/mL penicillin, and streptomycin (GIBCO, Grand Island, NY, USA). Cell cultures were maintained at 37 °C in a humidified atmosphere of 5% CO_2_. For the induction of primary cilia formation, cells were incubated under SD conditions in the absence of FBS.

### 4.3. Cell Viability Measurement

Cells were treated with vinblastine and/or appropriate reagents under various conditions. Cell viability was measured by MTT assay, trypan blue exclusion assay, or CellTiter Glo luminescent assay (Promega Co., Madison, WI, USA).

### 4.4. Preparation of Vinblastine-Resistant Cells

HeLa cells were incubated in the medium with 1 nM of VBL for 48 h. Dead cells were washed and VBL-resistant cells were incubated until confluent. Then, cells were sub-cultured for the second round of VBL treatment, and so on. Cells treated repeatedly with VBL three (3×), four (4×), five (5×), six (6×) or seven (7×) times were used for the following experiments.

### 4.5. Transfection of Nucleic Acids

Each plasmid DNA, siRNAs for NPHP3, and AccuTarget™ negative control siRNA were transfected into cells. NPHP3 was overexpressed by the transfection of cells with pCDNA6-NPHP3 or pEGFP-NPHP3 plasmid DNA, which was accompanied by pCDNA3.1 or pEGFP-C2 for the control group, respectively. Briefly, each nucleic acid and lipofectamine 2000 (Invitrogen, Carlsbad, CA, USA) or polyethylenamine (PEI) were diluted in a serum-free medium and incubated for 5 min, respectively. The diluted nucleic acid and lipofectamine 2000 reagent, or PEI, were mixed by inverting and incubated for 20 min to form complexes. Meanwhile, the cell culture medium was replaced with DMEM without antibiotics and FBS at least 2 h prior to the transfection. Pre-formed complexes were added directly to the cells, and the cells were incubated for an additional 6 h. Then, the culture medium was replaced with DMEM including antibiotics and FBS. In the case of transfection using PEI, no replacement of culture medium was required at 2 h before and 6 h after the transfection. Cells were incubated for 24–72 h prior to each experiment.

### 4.6. Immunofluorescence Staining

Cells with the indicated condition were grown on the coverslip [29,30,31,32], and primary cilia were immunostained with antibodies that are reactive with Arl13b and Ac-tubulin since they are biomarkers for cilia. Briefly, cultured cells were fixed with a 4% paraformaldehyde (PFA) solution freshly prepared in PBS for 10 min. Then, cells were permeabilized with 0.1% Triton X-100 in PBS and stained with antibodies to Ac-tubulin and/or Arl13b. Primary cilia were visualized by the incubation with secondary antibodies, chicken anti-mouse IgG-Alexa 488 for Ac-tubulin, and goat anti-rabbit IgG-Alexa 568 for Arl13b. Nuclei were visualized by staining cells with DAPI. Cells were observed and photographed at 400× or 1000× magnification with 40× or 100× objectives, respectively, under a fluorescence microscope (Nikon, Tokyo, Japan).

### 4.7. Gaussia Luciferase Assay for Promoter Activity

Pre-designed promoter (pmt) clones for NPHP3 (NM_153240) and IFT88 (NM_175605) in the pEZX-PG02 gaussia luciferase (Gluc) reporter system were obtained from GeneCopoeia Inc. (Rockville, MD, USA). NPHP3-pmt (HPRM12542) was 1309 bp (−1311~−3) upstream from the starting codon for NPHP3 transcription in Homo sapiens 3 BAC RP11-39E4 (AC055732.16). IFT88-pmt (HPRM46777) was 1401 bp (−2309~−645) upstream from the starting codon for IFT88 transcription in the Human DNA sequence from clone RP11-476H16 on chromosome 13 (AL590096.16). Schematic figures and sequences of promoters are shown in Supplementary Figures S4 and S5.

The Gluc activity of each reporter protein was measured as follows: Briefly, HeLa cells were transfected with the NPHP3-pmt-Gluc or IFT88-pmt-Gluc plasmids using PEI as described above. Then, cells were incubated for the appropriate time. Secreted Gluc reporter protein was collected from culture-conditioned media at indicated time intervals. Gluc activity of reporter protein was measured by using BioLux^®^ Gluc assay kit (New England BioLabs, Ipswich, MA, USA), including coelenterazine as Gluc substrate according to the manufacturer’s protocol. Luminescence was measured by using a Lumet 3, LB 9508 tube luminometer (Berthold Technologies GmbH & Co. KG, Bad Wildbad, Germany).

### 4.8. Reverse Transcription Polymerase Chain Reaction (RT-PCR)

Total RNA from culture cells was extracted by using TRizol reagent (Invitrogen, Carlsbad, CA, USA). Complementary DNA (cDNA) was synthesized by using 1 μg of isolated RNA, oligo-dT_18_, and superscript reverse transcriptase (Bioneer, Daejeon, Republic of Korea) in a total volume of 20 μL. Then, PCR amplification with 1 μL of template cDNA and with Taq DNA polymerase was performed with 25~35 thermocycles for 30 s at 95 °C, 30 s at 55 °C, and 60 s at 72 °C using human (h) oligonucleotide primers specific for each target gene, hNPHP3 (sense: AGC GAA ATA CCA AGC AAT GG; anti-sense: TGG AAG GTT CAC TTC CCA AG), hIFT88 (sense: AGT TCC AAG TGT CAA TAA GC; anti-sense: TTC TCG GTC TCC AAT AGC), hAATF (sense: GAC ACG GAC AAA AGG TAT TGC G; anti-sense: AGA CCC AGT CCC TCT GAA TCT), hGAPDH (sense: GAA GGT GAA GGT CGG AGT C; anti-sense: GAA GAT GGT GAT GGG ATT TC). PCR products for each gene were separated by 1.0~2.0% agarose gel electrophoresis and detected on the Ugenius 3^®^ gel documentation system (Syngene, Cambridge, UK).

### 4.9. Chromatin Immunoprecipitation (ChIP) Assay

The ChIP assay was performed by the method reported previously [50,51]. Cells for the ChIP assay were crosslinked with a final concentration of 1% formaldehyde for 10 min at room temperature. Then, unreacted formaldehyde was quenched with a 125 mM glycine solution. Cells were collected and applied to a Bioruptor (Cosmo Bio Co Ltd., Tokyo, Japan) to make DNA fragments with a size range of 200~1000 bp. Cell extracts were immune-precipitated using 1 μg anti-AATF antibodies or rabbit IgG control (Abcam, Cambridge, UK) for each sample diluted in 450 μl ChIP dilution buffer (0.01% SDS, 1.1% Triton X-100, 1.2 mM EDTA, 16.7 mM Tris-HCl, pH 8.1, 167 mM NaCl).

PCR analysis for all ChIP experiments was performed by using multiple sets of primers spanning on the NPHP3 or IFT88 gene promoter. Primers for the ChIP assay were obtained from pre-designed promoter sequences for NPHP3 and IFT88 provided by GeneCopoeia Inc. (Rockville, MD, USA). NPHP3 promoter (HPRM12542) was 1309 bp (−1311~−3) and IFT88 promoter (HPRM46777) was 1665 bp (−2309~−645), which were upstream from starting codon, ATG, of coding sequence (CDS) for NPHP3 and IFT88 transcription, respectively. Sequences for the primer set including the AATF binding site were 5′-CCC TGA TCC ACA TGG AGA ATT CA-3′ (forward) and 5′-CTT TAG ATT TTC TTC AGG AA-3′ (reverse), which corresponds to −1311 to −1112 bp on NPHP3 promoter and 5′-TAT ATT ACA TTG ATT AAG CTT TAC ACT ATG-3′ (forward) and 5′-CCT GAT GAA CTC ATC CGT AA-3′ (reverse), which corresponds to −1609 to −1260 bp on IFT88 promoter.

### 4.10. Western Blotting

The cellular protein level was detected by Western blot analysis. Briefly, cells were lysed in ice-cold lysis buffer containing 1% NP-40, a protease inhibitor (2 μg/mL aprotinin, 1 μM pepstatin, 1 μg/mL leupeptin, 1 mM phenylmetylsufonyl fluoride (PMSF), 5 mM sodium fluoride (NaF) and 1mM sodium orthovanadate (Na_3_VO_4_)). The protein concentration of each sample was measured by using a SMART^TM^ BCA protein assay kit from iNtRON Biotech Inc (Seoul, Republic of Korea). The same amount of protein in the sample buffer was heat-denatured, separated in sodium dodecyl sulfate polyacrylamide gel electrophoresis (SDS-PAGE), and transferred to the nitrocellulose membrane. An equal amount of loaded sample on the membrane was verified by ponceau S staining. The membrane was incubated with a blocking solution (5% non-fat skim milk in Tris-buffered saline with Tween 20 (TBST)), and incubated with the specific primary antibodies. Then, horse radish peroxidase (HRP)-conjugated secondary antibody was used as a target-specific primary antibody. Target proteins reacted with enhanced chemiluminescence (ECL) solution (Dong in LS, ECL-PS250) were detected by X-ray film (Agfa healthCare, CP-BU new) or chemiluminescence imaging system Fusion Solo (VilberLourmat Deutschland GmbH, Germany). In addition, IRDye-labeled secondary antibodies, IRDye 680RD Goat anti-Rabbit IgG, or IRDye^®^ 680RD Donkey anti-Goat IgG were used for target-specific primary antibodies. Target proteins were detected with the Odyssey CLx imaging system (LI-COR Biotechnology, Lincoln, NE, USA).

### 4.11. Yeast Two-Hybrid Screening Assay

Yeast two-hybrid (Y2H) screening was performed by Panbionet Corp. (http://panbionet.com, accessed on 10 April 2019–25 July 2019, Pohang, Republic of Korea). Briefly, a yeast two-hybrid assay was performed by using GAL4 DNA-binding domain (BD)-fused NPHP3 (691st~1330th aa) as a bait and a hybrid library of the human HeLa cDNA activation domain (AD) as prey. NPHP3 bait was cloned into EcoR I/Sal I sites of the pGBKT vector containing the DNA binding domain of GAL4 (GAL4-BD). Library A and B were used for Y2H screening. The cDNA inserts in library A were cloned as Sal I/Not I fragments in pPC86 containing GAL4-AD. The cDNA inserts in library B were introduced into yeast strain AH109 with a Sma I-linearized pGADT7-Rec vector in 3 different frames. Each insert DNA was integrated into the pGADT7-Rec (*LEU2* as a selection marker in yeast) vector, respectively, by yeast homologous recombination. The AH109 yeast strain was co-transformed with the bait DNA and prey library vectors. Y2H screening of NPHP3 was performed in a yeast AH109 strain containing two reporters (*ADE2* and *HIS3*) that are under the control of different *GAL* promoters. Yeast transformants were spread on selection medium [SD-leucine, tryptophan, histidine, and adenosine (SD-LWHA)] that supports the growth of yeasts with bait and prey plasmids yielding proteins interacting with each other. After selecting yeast colonies on selection media based on the reporter genes. We test protein-protein interaction using two independent reporters with different types of GAL4-binding sites to reduce the possibility of picking up false positive candidates. In order to confirm the interaction, the prey part of DNA from candidates was amplified by PCR or by *E. coli* transformation, and then the amplified candidate prey was reintroduced into yeast with the NPHP3 bait plasmid or with a negative control plasmid. Prey’s gene in each positive colony was identified by running alignment of DNA sequence in NCBI blast.

### 4.12. Statistical Analysis

In all experiments, the significant difference between groups was evaluated by One-way ANOVA and Independent or Paired sample *t*-test. The *p* value of <0.05 or <0.01 was considered to be significant.

## Data Availability

Data are contained within the article.

## Supplementary Materials

The following supporting information can be downloaded at: https://www.mdpi.com/article/10.3390/ijms251910369/s1.
