# Supplementary material for: Vinblastine Resistance Is Associated with Nephronophthisis 3-Mediated Primary Cilia via Intraflagellar Transport Protein 88 and Apoptosis-Antagonizing Transcription Factor
Source: Int J Mol Sci. 2024 Sep 26;25(19):10369. doi: 10.3390/ijms251910369 (PMC11477320; doi:10.3390/ijms251910369)
Supplement: Supplementary file 1 [file ijms-25-10369-s001.zip › ijms-3195748-supplementary.pdf]

Vinblastine resistance is associated with nephronophthisis 3-mediated primary cilium by intraflagella transport protein 88 and anti-apoptotic transcription factor

Pham Xuan Thuy[a], Tae-Kyu Jang[a] and Eun-Yi Moon[a, *]

[a]Department of Bioscience and Biotechnology, Sejong University, Seoul 05006, Republic of Korea

Running title: Vinblastine resistance with NPHP3-mediated PC by AATF and IFT88

*Corresponding author

Eun-Yi Moon, Department of Bioscience and Biotechnology, Sejong University, 209 Neungdong-ro Kunja-Dong Kwangjin-Gu, Seoul 05006, Republic of Korea.

Tel: +82 2 3408 3768; Fax: +82 2 466 8768.

E-mail address: eunyimoon@sejong.ac.kr (E.Y. Moon)

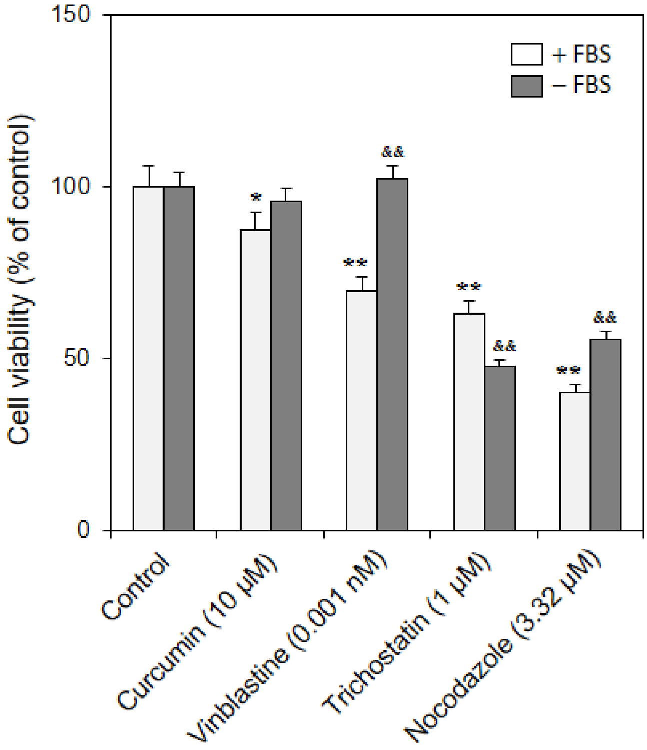

Supplementary Figure S1. Changes in cell viability by the treatment with various reagents, HeLa human cervical cancer cells were incubated with curcumin (10 μM), vinblastine (0.001 μM), trichostatin A (1 μM) and nocodazole (3.32 μM). Cell viability was measured by MTT assay. Each experiment was performed at least four times. Data in bar graphs represents the means ± SEM. *$p<0.05$, **$p<0.01$; significantly different from reagents-untreated control group with 5% FBS. $^{\&\&}p<0.01$; significantly different from each reagent-treated group with 5% FBS.

A

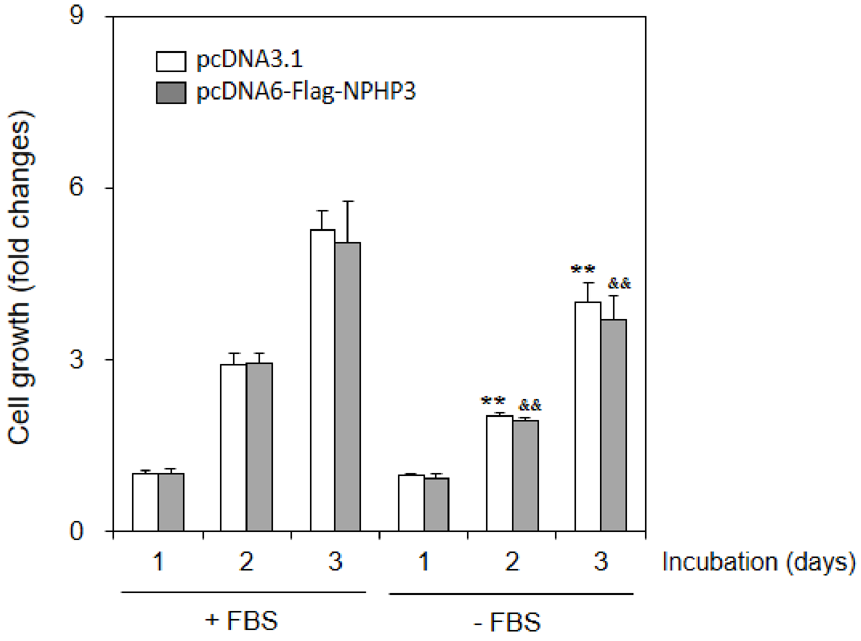

pcDNA3.1
pcDNA6-Flag-NPHP3
Cell growth (fold changes)
9
6
3
0
1
2
3
1
2
3
Incubation (days)
+ FBS
- FBS
**
&&
**
&&

B

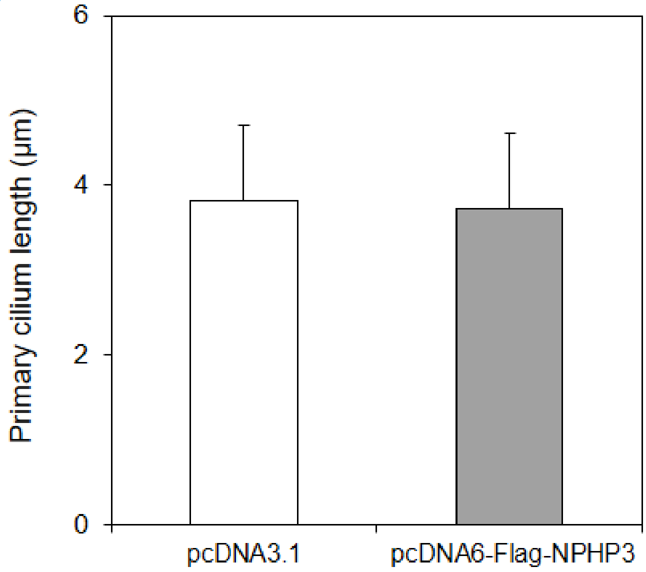

Primary cilium length (μm)
6
4
2
0
pcDNA3.1
pcDNA6-Flag-NPHP3

Supplementary Figure S2. No changes in cell proliferation and primary cell length was observed in the group with NPHP3 overexpression. (A, B) HeLa cells were transfected with pcDNA3.1 or pcDNA6-Flag-NPHP3 for 24 h. Each group was incubated for 3 days and total cell number was measured by using hemocytometer under brightfield microscope (A). The cells were fixed and stained with antibody against Ac-tubulin (green) and Arl13b (red). Nucleus was stained with DAPI (blue). The primary cilium was observed with 1,000X magnification under fluorescence microscope. Each primary cilium length was estimated by using ImageJ (version 1.54g). Data in bar graphs represents the means ± SEM. $^{**}p<0.01$; significantly different from pcDNA3.1-transfected control group with 5% FBS at each incubation day. $^{\&\&}p<0.01$; significantly different from pcDNA6-Flag-NPHP3-transfected control group with 5% FBS at each incubation day.

A

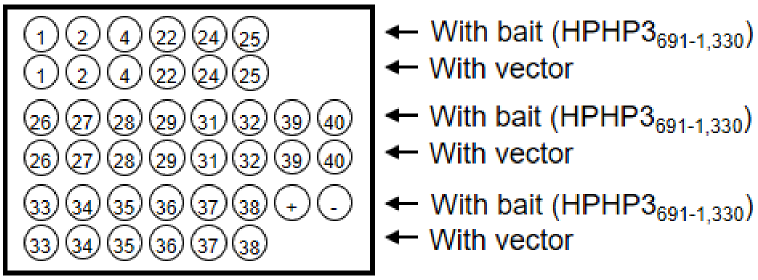

+, positive control (pGBKT7-p53 + pGADT7-Tag)
-, negative control (pGBKT7 + pGADT7)
number in circle, each prey bound AD

B *ADE2/HIS3* expression (SD-LWHA) Master plate (SD-LW)

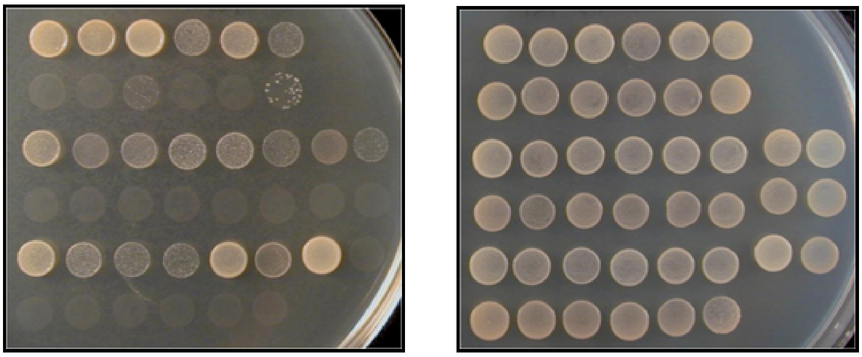

C

#	Gene (Homo sapiens)	NCBI number
1~3	SERTA domain containing 1 (SERTAD1), mRNA.	NM_013376
4~21	SERTA domain containing 1 (SERTAD1), mRNA.	
5	SERTA domain containing 1 (SERTAD1), mRNA.	
22, 23	Ring finger protein 31 (RNF31), transcript variant 1, mRNA	NM_017999.5
24	cDNA FLJ16199 fis, clone CTONG1000241, similar to RING finger protein 31 mRNA.	AK122711
26, 27, 28	BRCA1 associated ATM activator 1 (BRAT1), transcript variant 3, mRNA.	NM_001350627
29, 30	COP9 signalosome subunit 6 (COPS6), mRNA.	NM_006833
31	Nuclear receptor subfamily 1 group H member 3 (NR1H3), transcript variant 1, mRNA.	NM_005693
32	Farnesyl diphosphate synthase (FDPS), transcript variant 1, mRNA.	NM_002004.4
33	Mannosidase alpha class 1A member 2 (MAN1A2), mRNA.	NM_006699
**34**	**Apoptosis antagonizing transcription factor (AATF),mRNA.**	**NM_012138**
35	Ankyrin repeat domain 39 (ANKRD39), mRNA.	NM_016466
36	microtubule associated protein 1S (MAP1S), transcript variant 1, mRNA.	NM_018174
37	BCL2 like 12 (BCL2L12), transcript variant 1, mRNA.	NM_138639
38	laminin subunit gamma 3 (LAMC3)	NM_006059
39	DNA polymerase delta 1, catalytic subunit (POLD1), transcript variant X3	XM_005259008
40	Regulatory factor X associated ankyrin containing protein (RFXANK), transcript variant 3	NM_001278727

Supplementary Figure S3. Interaction of NPHP3 and anti-apoptotic transcription factor (AATF) by the yeast two-hybrid assay (Y2H). (A) The yeast two-hybrid assay with NPHP3 was performed in yeast AH109 strain containing reporters that are under control of different GAL4-AD fusion proteins. Baits were expressed as GAL4 DNA-BD fusion NPHP3 in the pGBKT plasmid; preys were expressed as GAL4-AD fusion proteins in pPC86 (#1 ~ 24) or pGADT7-Rec (# 26 ~ 40) vectors. (B) Yeast transformants of NPHP3 bait and GAL4-AD fusion proteins were spread on selection medium (SD-LWHA). The growth of #1 ~ 40 yeast colonies on selection medium indicated the interaction between NPHP3 and each prey protein. (C) Positive colonies were determined by DNA sequencing.

A

NPHP3 promotor (HPRM12542)

Length 1,309 bp

-1,311 ~ -3 from CDS

-1,234 ~ +74 from TSS

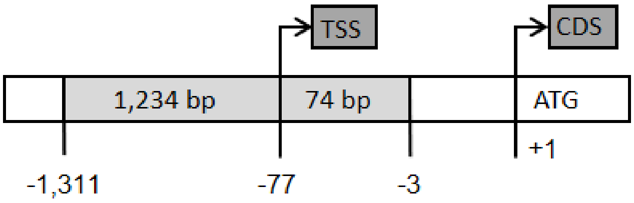

B

-1311 **c** **cctgatccac** **atggagaatt** **ca**aaaatttg aagttagaaa tcagaacgga

-1260 tttgaaaagg tagaaagttc ttaaggtact gtgatggtgg aaatatttcc ctttatctca

-1200 ggagaccacc tgaagaaatt agtatataca ctctggattt tgaagaatgc aaaggcctag

-1140 tatgactac**t** **tcctgaagaa** **aatctaaag**g gtaagagaag agatgactag ccactttgat

-1080 ggagggaatg aaggagagac tggtgtgcgg tctccaatgt cccatgccgc aatgagttaa

-1020 aaaacttaac cttgtctaag gattgtccct ggtggtttca gactgtaaaa tctaaaagct

-960 ctttctttgc tggcattatt ccttgctggt cattctatct acatgcatac tgagagagag

-900 atatcgcggg aagcacatag caggcactcc ataaattttt cagatttccc aactgcttcc

-840 agaaaggagt ttccggtcag gcacggcggc agcgaaagaa atagctgcag cgtttgtaga

-780 gcagctggga cccaggcctg tgctccctgt gaggcacgcc cgccagggag cctcgagccg

-720 ggcggagaca acggagagaa gccctatgtc ggagcaccac tccaccagcg ttcggagcac

-660 ccctccacca gcgtccggtc cactgctggt cctgcacagt cgccactccc cccatccttc

-600 ctgctagccc cgcccttctt gctagcccgc ctggccctgt gtgctttctc gacccacccc

-540 cgtttgccct gccccgccca catgccccgc cccacttgcc cggcccctct tgttgtcccg

-480 cctagccctg ctgccccgcc ccgtccccct cgcccatccc cccagttccg ctgccccgtc

-420 ccgttccgct gccccgcccc tccagttgcc ccacccccctt ccgctctccc tccccgcccc

-360 gttccgctgc ctcgcctcgt ccggctctcc ggccccgcccc cgccccgttc cgctgccccg

-300 ccccgtcctg ctccccccgcc ccgtcctgca cccccgcccc gcccagttct gctgccccgc

-240 ccctcctgtt tccccgcccc atcccgctcc cccgcctcgc cccgttccgc tgctccgccc

-180 cgtcccgctg ctccgcccag tcctgctctc cggccccgct ccatcccgct ggcccgcccc

-120 gtcccgttcc gttcctctgc cccgccccgt cccgttccgt tcc**t**ctgccc caccccgtcc

-60 cgttccgttc cgctgcccag tcctgctggt actcactagg tagtagcggc aacggacgcc

+1 ***atg***gggaccg cctcgtcgct cgtgagcccc gcgggcgggg aagtgatcga

Supplementary Figure S4. Schematic figures and sequences of pre-designed NPHP3 (NM_153240) promoter. (A) NPHP3 promoter (HPRM12542) was 1,309 bp (-1,311 ~ -3) upstream from starting codon, ATG, of coding sequence (CDS) for NPHP3 transcription. NPHP3 promoter covers 1,234 bp upstream and 74 bp downstream from transcription starting site (TSS). (B) Sequence of NPHP3 promoter includes TSS (black bold), and nucleotides (black underlined) to design primer sets for chromatin immunoprecipitation. Translation starting codon (black bold italic) is shown as underlined nucleotides.

A

IFT88 promotor (HPRM46777)

Length 1,401 bp

-2,309 ~ -645 from CDS

-1,401 ~ +263 from TSS

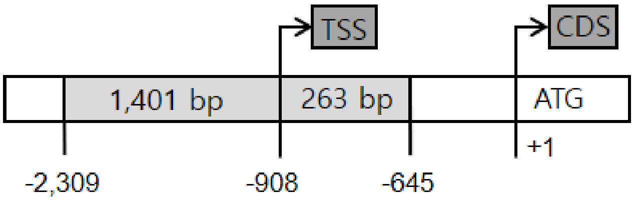

B

-2309 agctgaagg cagtctgggg gcaggattcc
-2280 ttcttcctca gaggacctta gtcttttctc ttatgacctt cccttgacgg gatgaggcca
-2220 acccaccttg tggaggacca actgctttac tcaagtctaa tttaactgtt aatcacatct
-2160 taaaaaatac cagctatcat agtactccag aacaaaggca gtcagtgtct ctgctcacaa
-2100 aacacgggaa agtgcaggtg acccatggag aattgtcccc caacactatg tttttctttt
-2040 ttttggatgg agtttcgctc ttgtcaccca ggctggagtg caataccacc atcttggctc
-1980 actgcaacct ctacctcctg ggttcaagag attctcctgc cttagcctcc tgagtagctg
-1920 ggatttatta caggcgcctg ccacctcgcc gggctaattt ttgtattttt agtagagaca
-1860 gggtttcacc acgttggcca ggctggtctc caactcctca cctcaggtga tccgccagcc
-1800 tcagcctccc aaagtgctgg gattataggc atgagccact gcgcccggcc aacactactt
-1740 attttataaa aactcattaa ttactggtgt ctttagactc cctctgtccc tcccctccat
-1680 ccccaacatt tacttttaaa atttaagaca aaacttaaac attaaacaat tatgaattta
-1620 attcccccat t<u>**tatattaca**</u> <u>**ttgattaagc**</u> <u>**tttacactat**</u> <u>**g**</u>acccaggtt ctaggtggtt
-1560 gggacacaat ggttcaatcc ctgtctagta aataaaacgg acaccttaag cgctacaatc
-1500 aaggtgcgca cggggtgcta cagaacaata cgcagacagg aggaggtggg gggaaaggtg
-1440 agacttccca ggcaggtgtc atcccagaat aacttttaca cagggtgact aagcgagtta
-1380 gtgactgcc ggaaaacggg cttccaaggt tcacaaggct cgtgctgcgg ctccgggagt
-1320 tatgtcacag taagcttact atcatccttt gggcatctgc t<u>**ttacggatg**</u> <u>**agttcatcag**</u>
-1260 <u>**g**</u>atttaaagg atcttggttc catatccttc cccttcctca cagaggccgc cagcccggag
-1200 cccctctagg ccctcctccc tcctgcatct actggccgcg agcctttccc tccccgcccc
-1140 cttcacacag gccgccccca gcctcccaac cccccggttc cgttccacgg gaggccccgg
-1080 cctccctgcc ctctcctcca ccgttctacc cgcatcgccc ggcttcccgc aagccgctgg
-1020 caccgtcccc tcaacaccct ccgcccaccg ccactccctt ccactgaggg ggaccgggct
-960 gccttctctc tgacccgccg ttctccgccc ccacccactt ccccaggcct cc**g**gcaccac
-900 cccctcccg ccgcgctctc tcccgccccc gcgcttccgg ccccgcccgc ccccgcgcgg
-840 aggactgtgg gagcggcttc cttggattcc gcgcttggca acggctcggc gtggcgcttt
-780 ggccaaccgc tgcgtcgtcc ctgggcccga ataactgtcg cccgcttccc tcagcgtgag
-720 gtaggagaag ggcgctgggg cctagtgcct cagggccct ggcagccgtg cccctgcggc
-660 gtcctgtgtg gcgcat

....

+1 ***atg***aaattca caaacactaa ggtacaaatg

Supplementary Figure S5. Schematic figures and sequences of pre-designed IFT88 (NM_175605) promoter. (A) IFT88 promoter (HPRM46777) was 1,665 bp (-2,309 ~ -645) upstream from starting codon, ATG, of coding sequence (CDS) for IFT88 transcription. IFT88 promoter covers 1,401 bp upstream and 263 bp downstream from transcription starting site (TSS). (B) Sequence of IFT88 promoter includes TSS (black bold), and nucleotides (black underlined) to design primer sets for chromatin immunoprecipitation. Translation starting codon (black bold italic) is shown as underlined nucleotides.
